# The efficacy of negative pressure wound therapy in treating sacroiliac joint tuberculosis with a chronic sinus tract: a case series

**DOI:** 10.1186/s13018-015-0250-4

**Published:** 2015-08-06

**Authors:** Xiaobo Luo, Xiangyu Tang, Yuanzheng Ma, Yonggang Zhang, Shuzhi Fang

**Affiliations:** Department of Orthopedics, General Hospital of Chinese People’s Liberation Army (301 Hospital), No. 28 Fuxing Road, Beijing, 100853 China; Department of Orthopedics, The 309th Hospital of Chinese People’s Liberation Army, Beijing, 100091 China; Chinese People’s Liberation Army 264 Hospital, Beijing, China

**Keywords:** Sacroiliac joint, Tuberculosis, Negative pressure wound therapy, Chemotherapy, Sinus

## Abstract

**Objective:**

Tuberculous sacroiliitis with abscess accounts for approximately 50 % of all sacroiliac joint tuberculosis cases. Tuberculous abscesses spread into the sacroiliac joint capsule, subcutaneous tissue, and the skin, and finally becomes a skin sinus. As there are no previous reports about sacroiliac joint tuberculosis with a chronic sinus, we evaluated its clinical characteristics and management by negative pressure wound therapy.

**Methods:**

A retrospective analysis of 12 patients with sacroiliac joint tuberculosis with chronic sinuses treated between January 2005 and January 2010 was conducted. Patients were treated with negative pressure wound therapy (NPWT). Treatment was divided into three phases: control phase, standard dressing changes daily for 4 weeks; interphase washout period, dressing changes every 3 days for 1 week; and intervention phase, no dressing changes until minimal sinus tract drainage (<5 ml per 24 h). Outcomes including the sinus healing time and the drainage volume were evaluated.

**Results:**

The mean follow-up was 37.1 months. Sinus healing was observed at an average of 25.25 ± 7.23 (range, 20–42) days after initial treatment. The mean volume of drainage did not change during the control phase, but decreased from 29.17 ± 16.63 to 0.25 ± 0.87 ml in the intervention phase. The mean daily reduction of wound volume, erythrocyte sedimentation rate (ESR), and C-reactive protein (CRP) in the intervention phase was greater than in the control phase (*P* < 0.05). Anti-tubercular therapy was administered an average of 14.00 ± 2.95 (range, 12–18) months. ESR and CRP returned to normal within 3 months after the sinus closure. Bony fusion was observed in 5 (41.7 %) patients, and fibrous ankylosis in the other patients at last follow-up. All patients healed uneventfully.

**Conclusions:**

Early diagnosis of sacroiliac joint tuberculosis with a chronic sinus can be difficult. NPWT provides better healing of sacroiliac joint tuberculosis with a chronic sinus than standard dressing changes.

## Introduction

Tuberculosis is a global problem, with an estimated 8.6 million new cases and 1.3 million related deaths in 2012. About 12 % of newly diagnosed cases are in China, making China the second largest tuberculosis country [32].

Skeletal tuberculosis accounts for 3 to 5 % of all tuberculosis cases. Sacroiliac joint tuberculosis accounts for approximately 10 % of skeletal tuberculosis cases [5, 13, 28]. The formation of paraspinal tuberculous abscesses is seen in 50 to 75 % of spinal tuberculosis cases [7, 18, 26]. Kim [17] and Gao [11] reported similar results in that almost 50 % of patients with sacroiliac joint tuberculosis had gluteal and inguinal abscesses. In the late stages of infection, sacroiliac joint abscesses destroyed the capsule, spread into the adjacent subcutaneous tissues, and finally form sinus tracts. Treatment can be challenging. Traditional surgeries include curettage, curettage plus arthrodesis, and the modified Smith-Petersen arthrodesis method [11, 17]. Negative pressure wound therapy (NPWT) is an advanced wound treatment protocol developed in recent years. However, there are no reports of NPWT used to treat sacroiliac joint tuberculosis with a chronic sinus.

The aim of this study was to evaluate the clinical characteristics and efficacy of NPWT in the management of sacroiliac joint tuberculosis with a chronic sinus tract.

## Methods

The records of 12 patients with sacroiliac joint tuberculosis and a chronic sinus tract treated at our institution between January 2005 and January 2010 were retrospectively reviewed. The drainage volume and inflammatory markers were assessed before and after NPWT. Demographic data collected included age, sex, location of the sinus, and surgical history. Because the cases in this study were rare, the sample size was small. In addition, the disease was chronic and recurrent. Therefore, a self-control study of inpatients before and after intervention was used in order to reduce bias. There was a control period, a middle period, and intervention period. Erythrocyte sedimentation rate (ESR), C-reactive protein (CRP), wound volume, and drainage were compared between the control period and the intervention period in order to evaluate the effects of NPWT.

The diagnosis of sacroiliac joint tuberculosis was based on clinical history and symptoms, physical signs, laboratory tests, imaging studies, and pathologic examination. Tuberculin skin testing, acid-fast bacilli (AFB) smear, culture results, ESR, and CRP were used as conventional markers of infection. Plain X-ray, ultrasound, computed tomography (CT), and magnetic resonance imaging (MRI) were performed to detect bone-marrow edema, sequestrum, sclerosis, abscesses, sinus tracts, and bony fusion. Laboratory and imaging testing were routinely performed during follow-up.

Pathologic specimens of sinus tract caseous necrosis were evaluated before NPWT. Neutral Lowenstein-Jensen culture medium was used for *Mycobacterium tuberculosis* culture. Drug susceptibility testing was performed on Lowenstein-Jensen medium using the proportion method. Routine bacterial cultures and sensitivity testing were performed.

### Treatment algorithm

Patients received standard anti-tuberculosis therapy before and after admission, and the anti-tuberculosis therapy was not modified during admission. Systemic antibiotics were administered when mixed infection was diagnosed. The results of common bacterial susceptibility testing were reported after 1 week, and antibiotic treatment was initiated according to bacterial susceptibility testing.

The treatment was divided into three phases: control phase, standard dressing changes daily for 4 weeks; interphase washout period, dressing changes every 3 days for 1 week; and intervention phase, no dressing changes until minimal sinus tract drainage (<5 ml per 24 h). All patients underwent the whole treatment process.

**Fig. 1:**
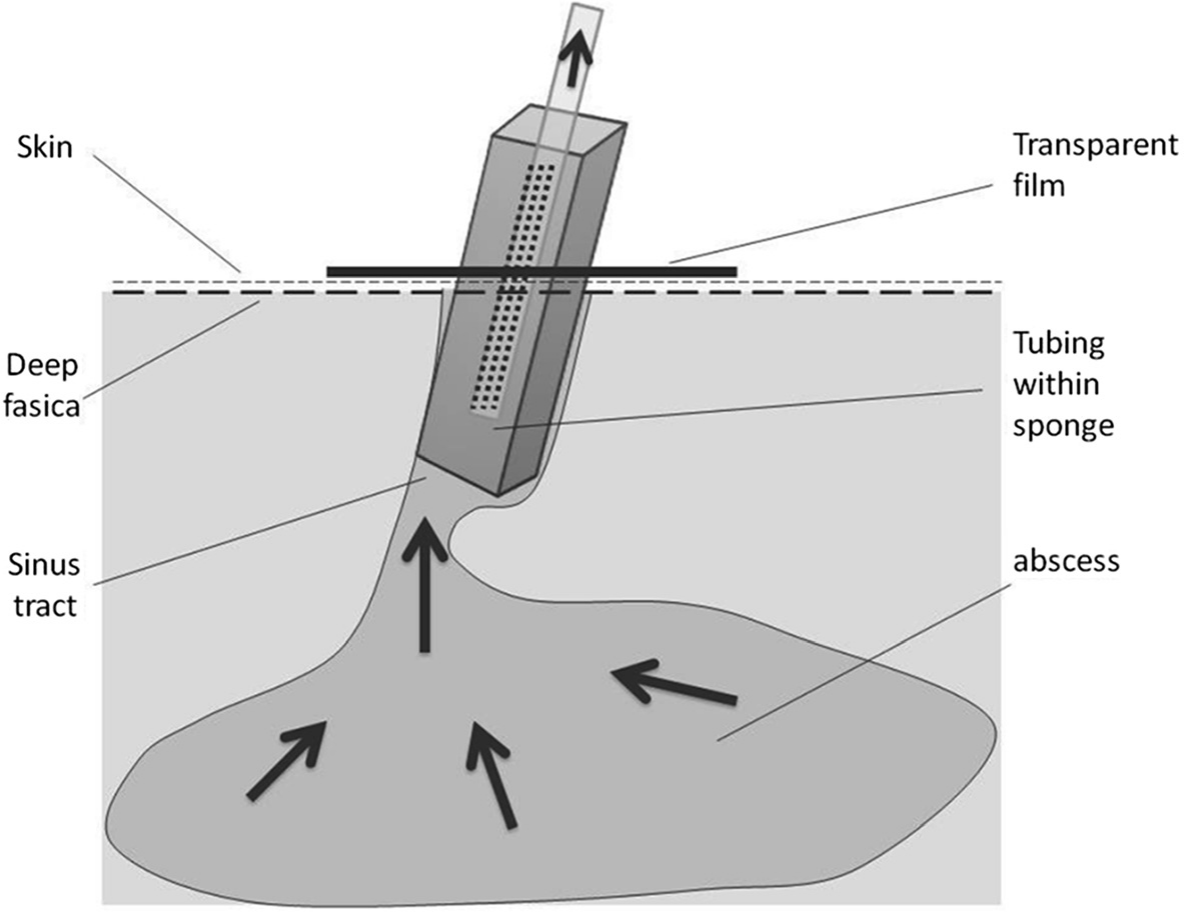
Schematic of the treatment

The NPWT procedure was performed at the bedside under local anesthesia. A suction tube wrapping by a medical sponge was inserted into the sinus tract, and a fluid-impermeable plastic film was used to seal the device. Tubing was attached to a vacuum pump at a pressure of −125 mmHg (Fig. 1). The vacuum device was used until granulation tissue formed, and little sinus tract drainage was present (<5 ml per 24 h).

After discharge, all patients continued anti-tuberculosis medications. Usually, the results of tuberculosis drug susceptibility testing were available after more than 2 months; drug therapy was modified according to the susceptibility result [19, 22].

Healing criteria included lack of pain or tenderness over the affected area, no discomfort during working, normal ESR, no abscess or sinus tract drainage, clearance and sclerosis of the joint margin, fusion of the sacroiliac joint, and the presence of fibrous ankylosis [17].

Clinical findings, imaging studies, ESR, CRP, and hepatic and renal function were routinely evaluated and recorded during and after admission. Fusion of the sacroiliac joint was assessed using plain X-ray, and CT and MRI were used to confirm bony fusion.

Statistical analyses were performed using SPSS 13.0 (SPSS Inc., Chicago, IL). Data were presented as number (%) and mean ± standard deviation (SD). Matched-pair *t* tests was used to evaluate significant differences between two phases. A two-tailed value of *P* < 0.05 was considered statistically significant.

## Results

There were seven female and five male patients included in the study. The average patient age was 32.8 years (range, 21 to 63 years), and the average length of follow-up was 37.1 months (range, 24 to 54 months).

All 12 patients complained of persistent buttock or low back pain, a chronic sinus tract, and difficulty walking. Ten (83.3 %) patients had typical symptoms of tuberculosis infection including weight loss, poor appetite, fever, and sleep hyperhidrosis. The pelvic compression test and Gaenslen’s test was positive on the affected side of all patients. Pain of the hip can be induced by hip over flexion and over extension. Four (33.3 %) patients had undergone abscess drainage or curettage before admissions. One (8.3 %) patient had bilateral sacroiliac joint tuberculosis, and seven (58.3 %) patients had left and four (33.3 %) had right sacroiliac involvement.

The sacroiliac joints of all patients were stable. Sinus tracts were found in all patients, including the buttocks in 11 (91.7 %), groin in 1 (8.3 %), and perineum in 1 (8.3 %). Eleven (91.7 %) patients had a single sinus tract, and 1 (8.3 %) patient had two. Five (41.7 %) patients had tuberculosis in other sites; two (16.7 %) patients had active pulmonary tuberculosis, and one (8.3 %) had infection at T10, L5, and in the intestine, respectively. Four (33.3 %) patients had prior surgery; three (25 %) had undergone abscess drainage and one (8.3 %) curettage. One (8.3 %) patient had a history of sacroiliac joint tuberculosis 16 years previously. No patient was infected with HIV (Table 1).

**Table 1:** Clinical features

Case	Sex/Age	Involved side	Other sites affected	Abscess	Sinus	Culture result/DST	Mixed infection
1	Female/24	Left	Lung	G	G	−/−	−
2	Female/34	Bilateral	–	I, G	G	+/−	−
3	Female/25	Left	T10	G	G	+/−	+
4	Female/60	Right	Lung	G	G	−/−	–
5	Female/46	Right	–	I, G	G	−/−	–
6	Male/23	Right	–	G	G	+/MDR	–
7	Female/33	Left	–	–	G	−/−	+
8	Male/26	Left	–	I, G	G	+/−	–
9	Male/51	Left	Intestine	G	G	−/−	–
10	Male/63	Right	L5	I, T	I	+/−	–
11	Female/21	Left	–	G	G	−/−	–
12	Male/42	Left	–	–	P, G	+/MDR	–

*G* gluteal, *I* inguinal, *T* thigh, *P* perineum, *AID* abscess incision drainage, *B* bone fusion, *F* fibrous ankylosis, *NPWT* negative pressure wound therapy

All patients were treated with NPWT according to the site and the size of the sinuses. The average duration of symptoms was 12.41 ± 4.36 months (range, 9 to 24 months). The average time interval between onset of low back pain and sinus tract formation was 8.50 ± 4.56 months (range, 3 to 20 months). The average duration of sinus tract drainage was 3.92 ± 1.08 months (range, 3 to 6 months). Patients received regular anti-tuberculosis therapy for a mean of 4.67 ± 1.37 months (range, 2 to 7 months) before admission. Patients were treated with NPWT for an average of 18.33 ± 6.97 days (range, 14–35 days). Sinus tract closure was observed after the initiation of NPWT at an average of 25.25 ± 7.23 days (range, 2042 days).

Culture in one (8.3 %) patient was positive for methicillin-resistant *Staphylococcus aureus* (MRSA) resistant to ampicillin, pefloxacin, ceftazidime, gentamicin, and ciprofloxacin. Vancomycin was used to treat this patient. One sinus culture revealed *Staphylococcus epidermidis*, and appropriate antibiotic treatment was initiated.

Fifty percent (6/12) of patients had positive cultures for *M. tuberculosis*. Of them, 33 % (2/6) were multi-drug resistant tuberculosis (MDR-TB). Of the six positive tuberculosis cultures, 33.3 % (2/6) were resistant to isoniazid, 33.3 % (2/6) to rifampicin, 16.7 % (1/6) to streptomycin, 16.7 % (1/6) to pasiniazid, and 16.7 % (1/6) were resistant to rifapentine. Resistance to three drugs was found in one case, and resistance to four drugs was found in one case. Anti-tubercular drugs were modified in two patients based on sensitivity testing after their wounds healed.

During the control phase, the duration of dressing changes was 30 days. The average daily drainage volume, wound volume, ESR, and CRP changed from 21.75 ± 8.86 to 21.33 ± 7.90 ml, 37.75 ± 33.80 to 35.17 ± 37.44 cm^3^, 40.16 ± 23.99 to 38.25 ± 21.63 mm/h, and 32.33 ± 12.09 to 33.33 ± 13.36 mg/dl, respectively. The average reduction of wound volume following treatment was 6.45 %.

During the 7 days of the interphase, the average daily drainage volume, wound volume, ESR, and CRP changed from 20.75 ± 6.94 to 22.33 ± 7.28 ml, 35.17 ± 37.44 to 34.08 ± 33.91 cm^3^, 38.25 ± 21.63 to 38.33 ± 20.82 mm/h, and 33.33 ± 13.36 to 33.67 ± 11.72 mg/dl, respectively. There was no significant difference of the change in the drainage volume, wound volume, ESR, and CRP in the control phase and interphase (*P* > 0.05).

**Fig. 2:**
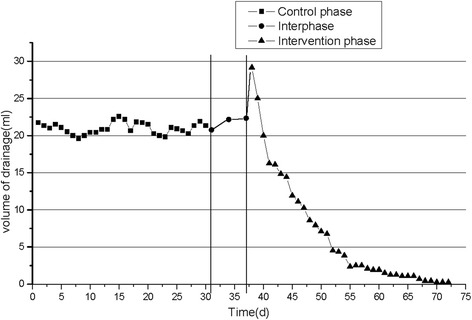
Daily drainage volume. *DC* dressing change, *NPWT* negative pressure wound therapy

**Fig. 3:**
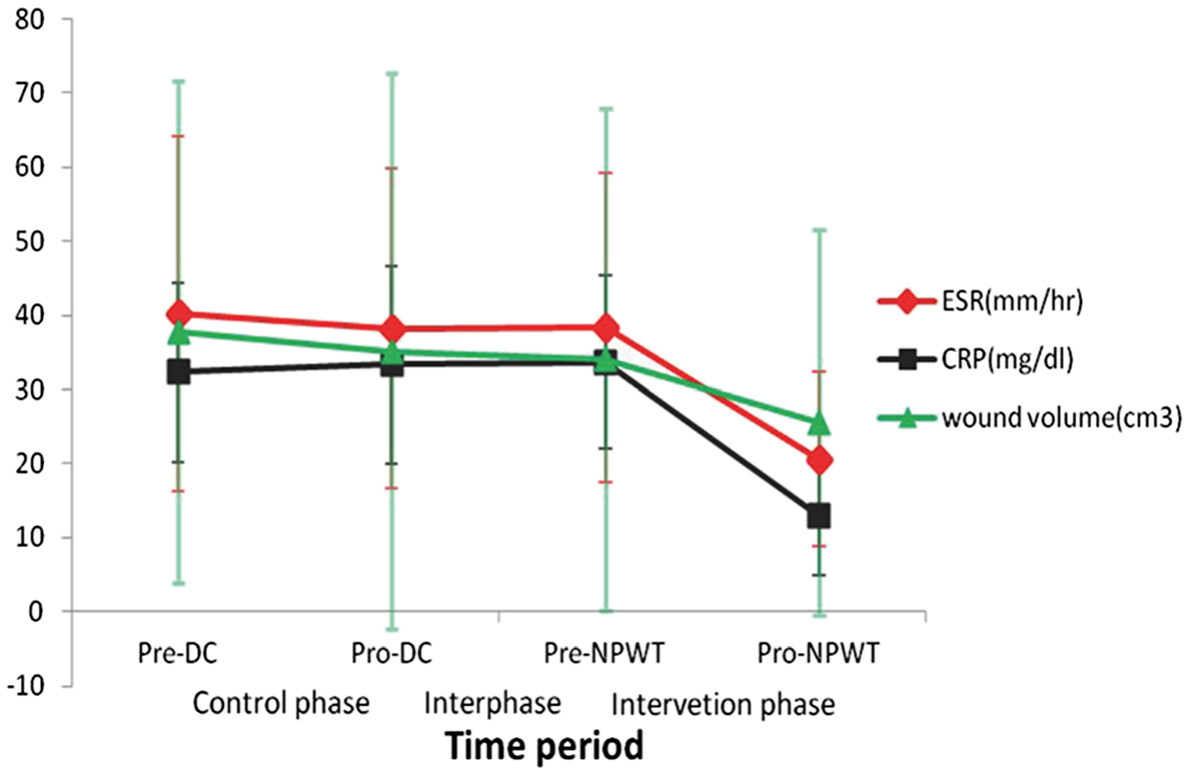
Comparison between mean ESR, CRP, and wound volume after admission. The data are expressed as mean ± standard deviation (*SD*)

In the intervention phase, average duration of NPWT was 18.33 ± 6.97 days (range, 14–35 days). The average drainage volume increased from 22.33 ± 7.28 to 29.17 ± 16.63 ml during the first 3 days. After that, the average daily drainage volume decreased from 29.17 ± 16.63 to 0.25 ± 0.87 ml on the 35th day. The average wound volume changed from 34.08 ± 33.91 to 25.50 ± 26.04 cm^3^ (*P* < 0.05). The average reduction of wound volume following the treatment was 26.98 %. The average ESR changed from 38.33 ± 20.82 to 25.50 ± 11.72 mm/h (*P* < 0.05). The average CRP changed from 33.67 ± 11.72 to 13.00 ± 8.01 mg/dl (*P* < 0.05). The reduction in average wound volume, ESR, and CRP were greater in the intervention phase than in the control phase (*P* < 0.05) (Figs. 2 and 3; Table 2).

**Table 2:** Wound volume, ESR, and CRP of patients who received dressing changes or NPWT

	Control phase (dressing changes)	Intervention phase (NPWT)	*P* value
Length of the treatment (days)	30	18.33 ± 6.97	
Pre-treatment wound volume (cm^3^)	37.75 ± 33.80	34.08 ± 33.91	0.061
Post-treatment wound volume (cm^3^)	35.17 ± 37.44	25.50 ± 26.04	0.019
Reduction of wound volume (cm^3^)	2.58 ± 2.74	8.58 ± 8.53	0.012
Pre-treatment ESR (mm/h)	40.16 ± 23.99	38.33 ± 20.82	0.32
Post-treatment ESR (mm/h)	38.25 ± 21.63	25.50 ± 11.72	<0.001
Reduction of ESR (mm/h)	1.92 ± 6.04	17.75 ± 28.82	<0.001
Pre-treatment CRP (mg/dl)	32.33 ± 12.09	33.67 ± 11.72	0.22
Post-treatment CRP (mg/dl)	33.33 ± 13.36	13.00 ± 8.01	<0.001
Reduction of CRP (mg/dl)	−1 ± 2.77	19.75 ± 32.56	<0.001

Data were presented as number (%) or mean ± standard deviation

*ESR* erythrocyte sedimentation rate, *CRP* C-reactive protein

**Fig. 4:**
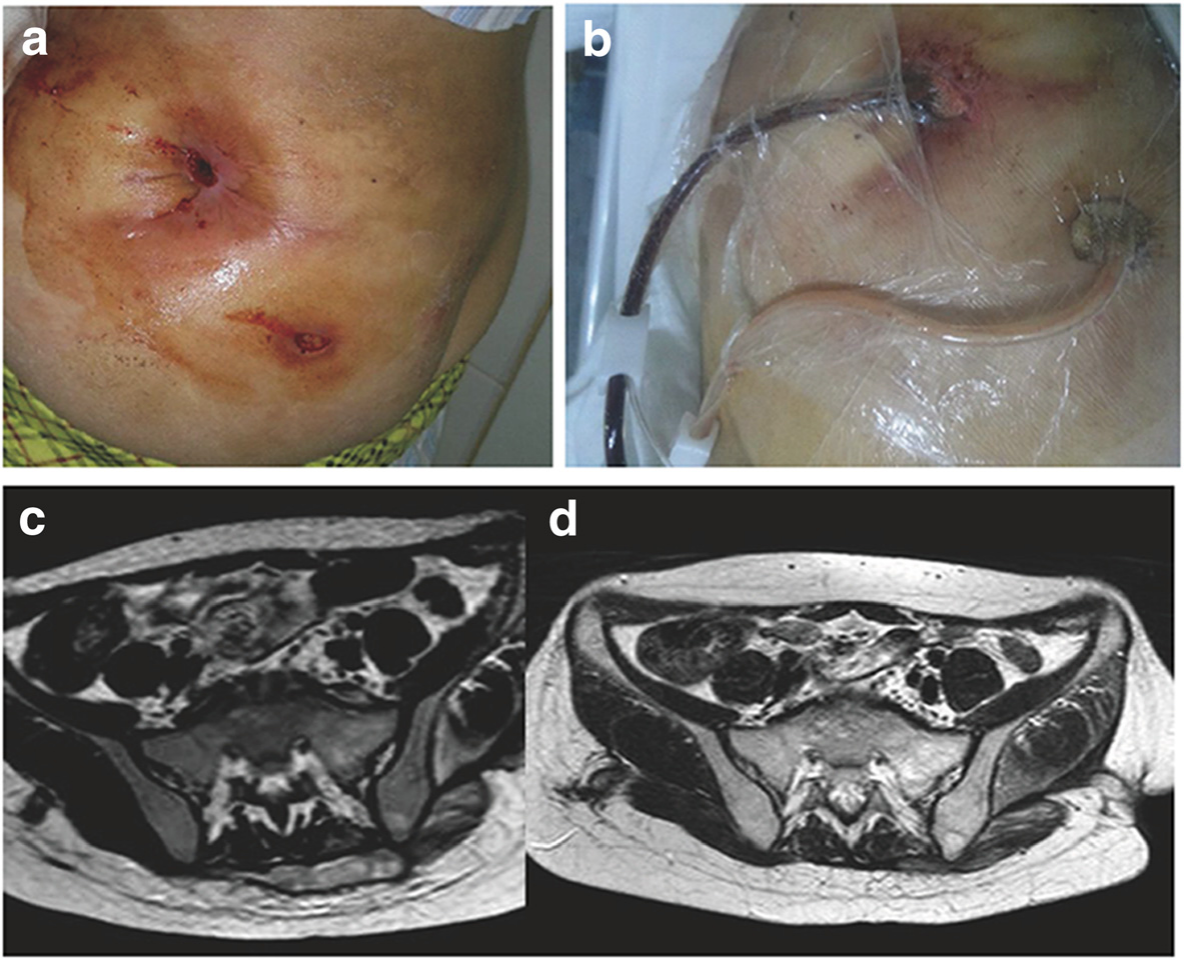
**a** Sinus tracts in the left buttock. **b** Management with NPWT. **c** MRI of the sacroiliac joints: erosive changes and abscess involving the right sacroiliac joint and sinuses in the left buttock. **d** MRI demonstrating healed abscesses and solid bony fusion 36 months after NPWT and anti-tubercular therapy

**Fig. 5:**
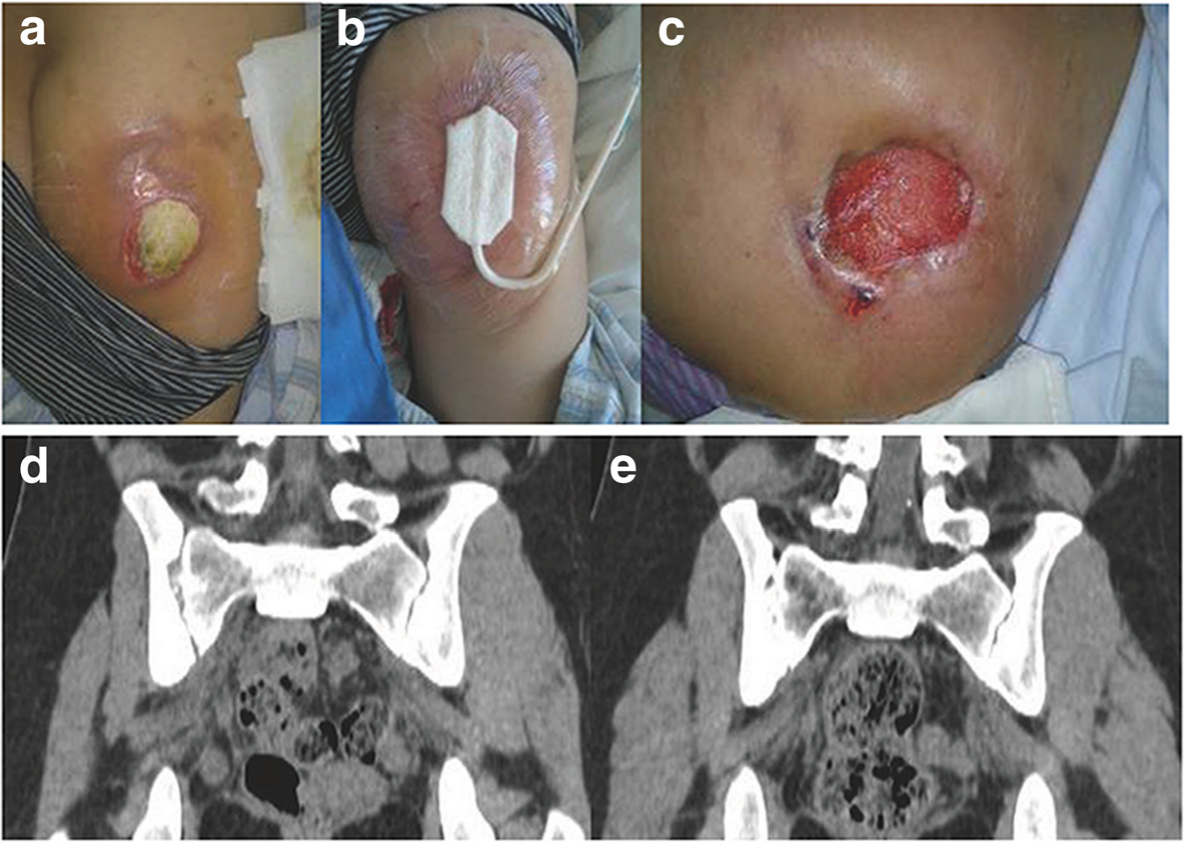
**a** Sinus and wound in the left buttock. **b** Management with NPWT. **c** Formation of fresh granulation tissue after NPWT. **d** CT scan of sacroiliac joints: erosive changes involving the right sacroiliac joint. **e** CT scan demonstrating bony fusion 30 months after NPWT and anti-tubercular therapy

ESR and CRP returned to normal by 3 months following discharge. Anti-tuberculosis therapy was administered for an average of 14.00 ± 2.95 months (range, 12–18 months). Definitive bony fusion was observed in five (41.7 %) patients and fibrous ankylosis in 7 (58.3 %) (Figs. 4 and 5). The average time to bony fusion was 41.20 ± 9.23 months (range, 30–54 months). All sinus tracts healed without recurrence. At final follow-up, one (8.3 %) patient had a little discomfort occasionally. No other complications occurred.

## Discussion

Sacroiliac joint tuberculosis is rare. The diagnosis of tuberculosis of the sacroiliac joint in the early stages is difficult due to the vague presenting symptoms, and thus the diagnosis is delayed in many cases [3, 11, 28].

Typical symptoms of the sacroiliac joint tuberculosis included groin or buttock pain, posterior thigh pain, and lower limb radiculopathy associated with walking. These findings may results in a misdiagnosis of arthritis or neurologic disease [14, 17, 24, 27, 28]. Radiographic imaging can be normal in the early stage of the disease. As the disease progresses, erosion of the sacroiliac joint becomes more distinct and abscesses appear.

**Fig. 6:**
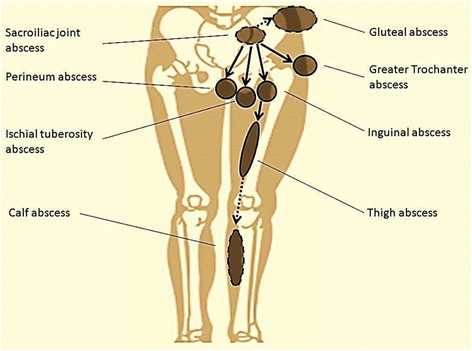
Typical paths of sinus tract drainage

The incidence of abscess formation is high in sacroiliac joint tuberculosis because the joint space is too narrow to accumulate a substantial amount of fluid. Rupture of an abscess through the skin forms a sinus tract. The abscesses are mainly restricted to the gluteal and inguinal areas, but because of the complicated anatomic structures adjacent to the sacroiliac joint, the formation of abscess can vary. Presacral abscesses may spread to the inguinal and iliac fossa along the iliopsoas muscle, and then extends into the thigh and calf areas. A presacral abscess can also spread to the perineum through the perirectal space, to the ischial tuberosity along the sacro-tuberous ligament, and to the trochanter along the piriformis muscle (Fig. 6).

The diversity of abscesses is always confusing and misleading. In addition to spontaneous abscess rupture, incision and drainage of an abscess can also lead to sinus tract formation. Gluteal or perineal sinuses formed spontaneously in eight (66.7 %) of our patients, and postoperative sinuses were found in four (33.3 %) patients; three after incision and drainage and one after curettage. We believe simple incision and drainage of tubercular abscesses puts patients at risk for sinus tract formation, and is therefore not recommended. Of our patients, 91.7 % (11/12) had gluteal sinuses and 8.3 % (1/12) inguinal sinuses. Gluteal sinuses were most commonly spontaneous because the gluteus muscle is compressed during sitting and lying down, thus facilitating local spread. Inguinal sinuses usually occurred after abscess incision and drainage.

NPWT is a non-invasive therapy applied to acute or chronic wounds of different etiologies. The concept of NPWT was first proposed by Fleischmann in 1993 [9]. NPWT was performed in our patients using a suction tube wrapped in foam, a fluid-impermeable transparent film, and a vacuum pump. This device is widely used in the treatment of acute and chronic wounds [1]. This method has also been used in the treatment of tuberculous mid-palmar abscesses, and tuberculous osteomyelitis of the sternum [10, 16]. However, there have been no studies examining NPWT in tuberculous wounds. All patients we treated with NPWT had a satisfactory outcome with no recurrence.

Advantages of NPWT may be the exhaustive debridement of pus, local improvement in tissue perfusion, mechanical traction and stimulation with negative pressure, decrease in bacterial levels [30], and reduction in local edema [23]. One reason for using NPWT to treat a tuberculous sinus tract is the decrease in the load of *M. tuberculosis* after applying NPWT. Some author considered the promotion of wound healing by NPWT may be explained by decreasing the bacterial load. One of the primary uses of NPWT therapy is the management of infected wounds.

Fleischmann et al. who was the earliest to propose NPWT therapy have also used the technique for the management of infected wounds [8]. Morykwas et al. constructed an animal model infected by a human isolate of *S. aureus* and a swine isolate of *S. epidermidis* and treated the infection with either NPWT or controlled moist saline dressing changes and found that NPWT resulted in a significant reduction in the wound bacterial load [20]. A similar result has also been seen in human wounds [31]. Many other studies have shown that NPWT assists in healing infected wound [6, 12, 15].

Although NPWT has been used to manage infected wounds successfully, some studies suggest that healing of contaminated acute and chronic wounds cannot be explained by a significant reduction in bacterial load [21]. In our study, all sinus tracts healed without recurrence after the initiation of NPWT at an average of 25.25 ± 7.23 days (range, 20–42 days). Wound volume and daily drainage volume decreased significantly after the application of NPWT. Thus, our results suggest that the positive effect of NPWT on tuberculous wound healing may be explained by a reduction of the bacterial load. However, the *M. tuberculosis* load was not determined because it was difficult to evaluate by with existing detection methods. Whether NPWT can reduce the *M. tuberculosis* load requires further study.

In this study, we evaluated wound healing during different stages of treatment, and tried to demonstrate the effective treatment of tuberculous sinus tracts using NPWT. There were no significant changes in drainage volume or wound volume during the control phase and interphase. However, in the intervention phase, drainage volume decreased significantly. This may be explained by exhaustive removal of pus, and the control of disease progression. In the intervention phase, the pre-treatment wound volume was not significantly different from that in the control phase (*P* > 0.05). But in the intervention phase, the post-treatment wound volume was 25.50 ± 26.04 cm^3^, and the reduction of wound volume was 26.98 %. These results were significantly different from the control phase (*P* < 0.05). Thus, NPWT can improve mechanical traction and granulation tissue proliferation in the tuberculous sinus tract. The mechanisms whereby NPWT promotes healing of the tuberculous sinus tract may include (1) reduction in the mycobacterium load to slow the progression of the wound and stop transmission, (2) persistent removal of the pus and necrotic tissue, and (3) promotion of granulation tissue proliferation. Moreover, NPWT decreased the hospitalization time and the number of daily dressing changes. In this study, all sinus tracts healed without recurrence. Thus, we believe the use of NPWT in tuberculous sinus tracts can also prevent relapse.

The ESR test determines the rate of fall of red blood cells in a column of anti-coagulated blood in 1 h [4]. CRP, an acute phase protein, is synthesized by hepatocytes in response to pro-inflammatory cytokines, in particular interleukin (IL)-6 [29]. ESR and CRP, which will initially be elevated significantly, and normalize over time on therapy, in patients with active tuberculosis have been used to predict disease severity and curative effects for several decades. Normalization of the ESR and CRP level are associated with resolution of the systemic inflammatory process and clinical response [2, 17, 25]. In our study, the pre-treatment ESR and CRP in the intervention phase were not significantly different from the control phase (*P* > 0.05). This study also showed that reductions of ESR and CRP in the intervention phase were significantly higher than those in control phase, and that NPWT for an average of 18.33 ± 6.97 days was associated with significant reductions of ESR and CRP.

## Conclusions

NPWT helped to make the treatment and nursing care simple, easy, and effective. The mechanisms whereby NPWT promotes healing of the tuberculous sinus tract may include (1) reduction in the mycobacterium load to slow the progression of the wound and stop transmission, (2) persistent removal of the pus and necrotic tissue, and (3) promotion of granulation tissue proliferation. The use of NPWT assists in the healing of sacroiliac joint tuberculosis and sinus tracts more effectively that dressing changes. Further studies are needed to confirm this conclusion.
